# Civic Engagement and Personality: Associations with the Big Five and the Dark Triad

**DOI:** 10.3390/ijerph20032126

**Published:** 2023-01-24

**Authors:** Pilar Rico-Bordera, José A. Piqueras, Victoria Soto-Sanz, Tíscar Rodríguez-Jiménez, Juan-Carlos Marzo, Manuel Galán, David Pineda

**Affiliations:** 1 Forensic Psychology Unit of the Centre for Applied Psychology, Miguel Hernández University of Elche, 03202 Alicante, Spain; 2 Department of Health Psychology, Miguel Hernández University of Elche, 03202 Alicante, Spain; 3 Department of Psychology and Sociology, University of Zaragoza, 44003 Teruel, Spain; 4 Department of Psychology, Faculty of Medicine, Catholic University of Murcia, Guadalupe de Maciascoque, 30107 Murcia, Spain

**Keywords:** Dark Triad, Big Five, civic engagement, personality, narcissism

## Abstract

Several studies have analyzed the relationship between general personality traits and attitudes and behaviors, indicating that a person is more committed to the community. After raising the question of whether malevolent traits might also be related, the aim was to analyze the relationship between civic engagement and personality, delving into the contribution of the Dark Triad (narcissism, Machiavellianism, and psychopathy) and controlling for the association with the Big Five. The Civic Engagement Questionnaire, the Short Dark Triad, and the Big Five Inventory-10 were administered to 1175 Spanish students (convenience sampling). After performing statistical analyses using SPSS statistical software, it was obtained that the three Dark Triad traits explained 11% of the total explained variance of civic engagement, while 19% was reached when the Big Five were included. Narcissism and openness were the factors most strongly associated with engagement. The positive relationship between narcissism and general personality traits could explain why narcissistic people have more favorable attitudes. Furthermore, people with narcissistic traits may display these attitudes for their own benefit. This study provides further evidence of how the narcissistic personality trait differs from the other two malevolent traits. Given that these traits are also associated with maladaptive behaviors, knowing all their characteristics could facilitate the design of prevention programs aimed at reducing such maladaptive behaviors.

## 1. Introduction

Civic engagement is the attitude of believing that one can and should make a difference for the betterment of the community, requiring knowledge, attitudes, skills, and values to achieve that betterment [1,2]. It consists of promoting the quality of life of a community through civic behaviors (considering both political and non-political processes, given their relationship), which in turn has positive effects on citizens’ sense of community and their attachment to their place of residence [1,2,3,4].

The different authors who have considered civic engagement in their studies have done so from different approaches, analyzing it in a more specific way and asking about certain behaviors (for example, asking whether they pay taxes and vote in elections) or in a more general way (for example, asking whether they are committed to serving in their community) (e.g., [5,6,7]). These civic behaviors have been positively related to mental and physical health and well-being in people who engage in them, and, in turn, the sense of community has been related to social well-being [8,9,10].

Several studies have analyzed its relationship to broader personality traits to identify the main characteristics that are related to community engagement. The interest has been in analyzing which general traits are related to those attitudes and behaviors that indicate that a person is more engaged in their community [6,11,12,13,14,15]. On this matter, different studies have focused on the Big Five personality traits [16] and, to a lesser extent, the Big Six personality traits (HEXACO; [17]). These constructs include extraversion, agreeableness, conscientiousness, openness to experience, emotional stability (or neuroticism in the negative sense), and honesty–humility (the latter present only in the HEXACO).

Overall, there does not seem to be a consensus on which traits are related to civic engagement, as different relationships have been obtained in each study and the same traits are not always positively associated [6,11,12,13,14,15]. For example, some find positive relationships with all traits [11], and others find relationships with all traits except emotional stability [15], conscientiousness [12], or openness to experience [6]. Other studies, on the other hand, only find relationships with extraversion [14].

A cross-sectional study in 24 countries that examined the association of personality with political and civic participation found that the effects of the Big Five vary considerably across countries and that the results also depend on exactly which variables are measured. Furthermore, they consider that the effects of the Big Five on participation may also be mediated or moderated by other variables yet to be studied [7]. Perhaps, for this reason, different relationships were obtained in the studies mentioned above.

The question of the relationship between civic participation and malevolent (or socially undesirable) personality traits remains. Are there people who, despite having malevolent traits, are also committed to their community? So far, only one study has been located that has analyzed the relationship between attitudes towards good citizenship and civic duty (being a good citizen) and malevolent traits. This is the study by Pruysers et al. [6], who set out to extend the analysis beyond general personality traits to include dark traits. To do so, using a cross-sectional analysis (with regression models), they considered in their analyses both general traits (HEXACO), and Dark Triad traits in a sample of 371 Canadians (M_age_ = 49.20; SD = 15.20; 58% female).

The Dark Triad was defined in [18] by Paulhus and Williams and is composed of three malevolent personality traits: subclinical narcissism, Machiavellianism, and subclinical psychopathy. Broadly speaking, narcissism refers especially to grandiose identity and the need for admiration; Machiavellianism to lack of morality and manipulation of others; and psychopathy to callousness and impulsivity [19].

Therefore, considering these three traits and their relationship with civic engagement, Pruysers et al. [6] obtained statistically significant relationships for both narcissism and psychopathy, in a positive sense in the first case and in a negative sense in the second. Thus, they found that people with narcissistic traits are more engaged in the community, while people with psychopathy traits are less likely to be engaged. They did not find a significant relationship with respect to Machiavellianism.

These results, which indicate that people with narcissistic traits do actively engage with citizenship, question the meaning of narcissism since it is considered a malevolent or “dark” personality trait. In addition, the relationship between narcissism and other characteristics considered socially desirable, such as well-being or emotional intelligence, has also been examined and positive relationships have been found [20,21,22,23,24,25,26,27].

These relationships are interesting because it is important not to forget that Dark Triad traits have been associated with a wide variety of violent behaviors (bullying, sextortion, intimate partner violence, and cyberviolence or general delinquency, among others) [28,29,30,31,32,33]. Therefore, it is interesting to analyze why people with narcissistic traits do engage with their community. Pruysers and colleagues [6], for example, consider that people with narcissistic traits may seek praise and admiration from others and therefore perform these good deeds. In addition, it is well known that people with narcissistic traits constantly seek to boost their self-esteem and ego, characteristics that, in turn, overlap with other healthy characteristics in people, such as mental strength and lower stress levels, resilience, or be perceived as a good leader [34,35,36].

Taking these results into account, the recent interest of many authors has been in finding an answer to why narcissism behaves differently from the other two malevolent traits (Machiavellianism and psychopathy). For example, Van Groning et al. [22] consider that the presence of other traits or skills (considered socially desirable) confers on narcissism the characteristic of being a protective factor concerning the rest of the traits of the Dark Triad. Based on this idea, the positive relationship between civic engagement and subclinical narcissism could be explained by following this argument and considering that narcissism is at the same time correlated with other more general personality traits, even though some studies have concluded that the inclusion of the Dark Triad personality traits does not offer real predictive advantages over the HEXACO, which contemplates the honesty–humility variable [37,38,39].

However, following Weinschenk’s indications [7], the effect of the Big Five on civic engagement could also be influenced by other unknown variables. Furthermore, it is well known that narcissism is positively associated with some of the Big Five factors, generally, with extraversion, conscientiousness, or openness to experience, and negatively with the other two (i.e., with neuroticism and agreeableness). Moreover, these associations are somewhat different in comparison with the other two traits of the Dark Triad, i.e., Machiavellianism and psychopathy, since, in general, these seem to relate positively to neuroticism and negatively to conscientiousness, openness to experience, and agreeableness. In the case of extraversion, Machiavellianism seems to relate negatively and psychopathy positively [40,41,42,43,44,45,46,47]. Following this idea, the study by Pruysers et al. [6] is the only one located that has analyzed the relationship of civic engagement with both the traits considered to be more general and the traits considered to be more socially undesirable. However, there are no studies with any population, that have analyzed these relationships together, that is, that have considered in a single statistical model the associations of both general and malevolent traits with civic engagement. Therefore, no studies have considered the possible differences between the association with malevolent traits when they do so alone and when they do so together with more general traits. Moreover, Pruysers and colleagues concluded that further studies are needed to be able to generalize the results they obtained in their analyses with confidence since they were the first to look at the relationship between civic engagement and dark personality traits.

### The Present Study

As stated, there are currently no studies that have analyzed the relationship between civic engagement and personality traits, both general and malevolent, and that have considered analyzing the possible relation of some traits with others in determining this engagement (on the assumption that some are malevolent and should not be related to civic engagement).

Considering the few results in the literature and the controversy with the narcissistic trait, our main concern is to know the association of malevolent traits with civic commitment and to know if there are differences between the association with malevolent traits when they are the only traits included in the statistical model and when they are included together with the more general traits. Therefore, based on these ideas, this paper aimed to analyze the relationship between civic engagement and personality traits, delving into the specific contribution of the Dark Triad traits and controlling for the association with the Big Five personality traits in a sample of Spanish young adults.

Consistent with previous literature [6], we expect to obtain a significant association between civic engagement and narcissism and psychopathy (positive in the first case and negative in the second case), and to obtain a non-significant association with Machiavellianism (H1). In turn, taking into account the inconsistency of the literature and the difficulty in establishing a hypothesis [6,11,12,13,14,15], a significant association is expected between civic engagement and the Big Five traits (positive for all except neuroticism, which is expected to be negative) (H2). Finally, we expect to obtain differences in the magnitudes of association with civic engagement when malevolent traits are associated alone and when they are associated with more general personality traits (i.e., when they are included together in the same statistical model) (H3). The latter hypothesis is not supported by previous literature, as this is the first study to raise this question.

## 2. Materials and Methods

### 2.1. Sample

The sample consisted of 1175 students (683 females, 58.1%) from two Spanish universities (Miguel Hernandez University of Elche and San Antonio Catholic University of Murcia). The mean age was 20.51 years (SD = 2.52, range 17–30 years).

To determine the sample size (convenience sampling), we allocated several observations 6 to 10 times greater than the variables [48]. Accordingly, the sample needed size ranged between 264 and 440 participants, based on the number of items of the Civic Engagement Questionnaire, Big Five Personality Traits-10, and the Dark Triad. Finally, 1733 participants took part in the study. However, 558 cases had to be eliminated because they had not completed the online survey until the end. Therefore, in the end, the sample consisted of 1175 participants.

### 2.2. Measures

#### 2.2.1. Civic Engagement Questionnaire (CEQ)

The CEQ [49] is a subscale developed from the Positive Youth Development Inventory (PYDI; [50]). Based on seven items, it generally measures young people’s perception of their contribution to the community (e.g., it is important to me to try to do something to change the world or I like to work with others to solve problems). A six-point Likert-type scale from 1 = strongly disagree to 6 = strongly agree was used. In the present sample, it shows an adequate reliability index, with an acceptable alpha (Cronbach’s alpha = 0.78 and McDonald’s omega = 0.79), like that obtained in the original version.

#### 2.2.2. Big Five Inventory-10 (BFI-10)

The BFI-10 [51] is a shortened version of the 44-item BFI and measures the Big Five personality traits: extraversion (e.g., I see myself as someone who is outgoing, sociable), agreeableness (e.g., I see myself as someone who generally trusts others), conscientiousness (e.g., I see myself as someone who does a thorough job), openness to experience (e.g., I see myself as someone who has an active imagination), and neuroticism (e.g., I see myself as someone who gets nervous easily). Each factor contains two items and is answered on a 6-point Likert-type scale (from 1 = strongly disagree to 6 = strongly agree). The design of this instrument proved that the BFI-10 retains a substantial part of the reliability and validity of the BFI-44 [52]: good test–retest reliability, convergent validity with another scale (NEO-Personality Inventory-Revised; [53]), and external validity. For the present sample, the correlation between the items of each factor (obtained with Pearson’s correlational analysis) is as follows: extraversion = 0.66, agreeableness = 0.03, conscientiousness = 0.21, openness to experience = 0.31, and neuroticism = 0.50.

#### 2.2.3. Short Dark Triad (SD3)

The SD3 [19] is a 27-item self-report that measures the following malevolent personality traits of the Dark Triad: subclinical narcissism (e.g., people see me as a natural leader), Machiavellianism (e.g., make sure your plans benefit you, not others), and subclinical psychopathy (e.g., revenge must be swift and unpleasant). Each factor contains nine items and is answered on a Likert-type scale from 0 = strongly disagree to 4 = strongly agree. It has been validated with a Spanish sample and has presented adequate reliability indices in the present sample with an acceptable alpha (Cronbach’s alpha: narcissism = 0.64, Machiavellianism = 0.79, and psychopathy = 0.67), similar to that reported by the authors [54]. It also has an acceptable omega (McDonald’s omega: narcissism = 0.65; Machiavellianism = 0.79, and psychopathy = 0.71).

### 2.3. Procedure

Participants were recruited through institutional outreach and the survey was carried out using the online data collection platform DetectaWeb [55] during the months of October, November, December, and January of the 2017/2018 academic year. To carry out the study, the project received approval from the university’s ethics committee (Reference DPS.JPR.03.17) and followed the ethical standards outlined in the 1964 Declaration of Helsinki. All participants gave their consent to participate in the study.

When participants accessed the survey link from their cell phones, tablets, or computers, they were first shown the instructions, then asked for their consent to participate, and then filled in the different scales of the study, for which they needed approximately 20 min. Participants did not receive compensation for participating in the study.

At the time of the project, many more questionnaires were administered than those used for this study. We administered measures for mental and socioemotional health, positive and negative affect, level of distress, anxiety, and depression, emotional intelligence, avoidance and fusion, suicidality, internalizing and externalizing symptoms and prosocial behavior, self-esteem, quality of life, healthy behaviors, and sincerity. However, only variables measuring civic engagement, the dark triad, and the Big Five were considered as variables of interest for this study. Consequently, the present study was part of a larger study in which all the measures described above were tested.

### 2.4. Design

A cross-sectional study (descriptive-correlational) was designed for the study. First, the descriptive statistics and the scores of the sample in the different questionnaires administered were calculated to obtain the profile of the participants and the mean of the different scores. The internal consistencies of the Civic Engagement Questionnaire and the Short Dark Triad were also estimated by calculating Cronbach’s alpha and McDonald’s omega coefficients following the recommendations of Kalkbrenner [56] for instrument reliability.

Secondly, to test the first two hypotheses of the study (H1 and H2), the correlations between the different variables under study were calculated to delimit the magnitudes and the positive or negative direction of the relationships between the different variables. For this same purpose, a multiple regression analysis was conducted to determine the associations of both dark traits and general personality traits with civic engagement (criterion variable). For this purpose, and to test H3, first, the specific contribution of gender as a sociodemographic variable (first block) was considered. Age was not included in the model due to the small variance in the sample (limited range of 17 to 30), anticipating that its effect would be null; in the second block the three Dark Triad traits were considered; and in the third block, the Big Five personality traits were considered. The percentage of total variance explained (*sr*²) for each of the variables was also calculated.

Given the large sample size, correlations and magnitudes of association in the regression model were interpreted after Bonferroni correction to obtain more accurate results (a significant effect was *p* < 0.0056, because of dividing the alpha (0.05) by the number of analyses performed, i.e., nine). Data were analyzed using SPSS (The Software IBM SPSS, 2021) and Jamovi (The jamovi project, 2021) statistical software. This study’s design and its analysis were not pre-registered.

The Spanish version of the Big Five Inventory-10 and Civic Engagement Questionnaire were adapted into the Spanish language in accordance with the guidelines of the International Test Commission [57], using an iterative-translation method that began with several independent translations. The item translations were then reviewed by a joint committee of translators with knowledge of the Spanish language and culture and specialists in the field of psychological assessment who analyzed the adequacy of the adapted version. To be sure that all items were well understood for young people, interviews asking about comprehension of the items were performed.

## 3. Results

### 3.1. Descriptive Statistics of the Instruments

The descriptive statistics of the instruments are presented in Table 1. In relation to the civic engagement, the mean score is moderately high. For the three dark traits (Dark Triad) participants seem to score higher on narcissism and Machiavellianism, and in the case of the Big Five scores, these are quite similar across the different factors, but they score higher on openness to experience.

### 3.2. Correlations between Civic Engagement, Dark Triad Traits, and the Big Five Personality Traits

The correlations (with the Bonferroni fit) between the different variables of interest for testing the first two hypotheses of the study (H1 and H2) are presented in Table 2. Civic engagement correlates significantly with two of the Dark Triad traits: narcissism and Machiavellianism. However, in the case of narcissism, it correlates positively and in the case of Machiavellianism it correlates negatively. In turn, civic engagement is significantly positively related to four of the Big Five personality traits: extraversion, agreeableness, conscientiousness, and openness to experience. In the case of neuroticism, the relationship is negative, but also significant.

The relationship between the Dark Triad traits and the Big Five is more heterogeneous. Narcissism correlates significantly with all five traits, but positively with extraversion, conscientiousness, and openness to experience, and negatively with agreeableness and neuroticism. Psychopathy is only significantly and negatively related to agreeableness and conscientiousness, and Machiavellianism is negatively related only to agreeableness.

### 3.3. Associations between Civic Engagement, Dark Triad Traits, and the Big Five Personality Traits

The regression model (with the Bonferroni fit) testing the three hypotheses of the study is presented in Table 3. In relation to the socio-demographic variable (gender; first block), a null contribution (0%) of the total explained variance of civic engagement was observed. However, when the three traits of the Dark Triad were included in the model (second block), a contribution of 11% (*p* < 0.001) was observed, which reached 19% (*p* < 0.001) when the Big Five were included as a third step (third block).

More specifically, in the second block, all three dark traits were found to be significantly associated with civic engagement (*p* < 0.0056, Bonferroni fit), but when the Big Five were introduced into the model, only narcissism remained significant (positive relation). In turn, of the Big Five, only agreeableness, conscientiousness, and openness to experience presented a significant (and positive) association (*p* < 0.0056, Bonferroni fit). In that third block (with all traits included in the model), it was observed that of the Dark Triad traits, narcissism was the factor with the highest specific contribution (*sr*² = 3.42%). In the case of the Big Five, openness to experience was the factor with the highest specific contribution (*sr*² = 4.37%).

## 4. Discussion

This study started from the question of whether people with malevolent traits were also engaged in the community and, having looked at the results of the only localized study that had analyzed these relationships [6], asked why people with narcissistic traits did appear to have civic behaviors (as opposed to the other two malevolent traits). Furthermore, considering the possible influence of other variables on these relationships [7,22], the possibility was raised that the Big Five might modify these relationships. Therefore, the aim of this study was to analyze the relationship between civic engagement and personality traits, delving into the specific contribution of the Dark Triad traits and controlling for the association with the Big Five personality traits in a sample of Spanish young adults.

Firstly, descriptive analyses have shown that the participants’ scores on the different questionnaires are within the mean compared to other studies [8,30,52,54]. Moreover, considering the ranges of the different scales, the scores are neither too high nor too low.

Secondly, to test the first two hypotheses of the study (H1 and H2), a correlational analysis and a regression model were conducted to test the associations between civic engagement and personality traits, both general and malevolent. The results showed the null association of the socio-demographic variable of gender with civic engagement, contrary to what was found in previous studies [6,11,12]. This could be due to differences in the characteristics of the samples in terms of culture and age (in the present sample, the average age is lower than in the other studies: 20.51 versus 50), although Doolittle and Faul [5] pointed out in their study that there is no theoretical basis to support the idea that age and gender influence civic attitudes.

On the one hand, in relation to the three traits of the Dark Triad, the (positive) association of civic engagement with narcissism stands out, being weaker than the (negative) association with Machiavellianism and psychopathy, when only the Dark Triad is included in the model. Thus, H1 is only partially confirmed, since we expected to obtain significant associations only with narcissism (positive) and psychopathy (negative) and, therefore, a non-significant association with Machiavellianism.

These results do not coincide with those obtained in the only localized study that has analyzed these associations using a regression model, since that study did not obtain a relationship with Machiavellianism [6]: Pruysers et al. considered that the motivations and interests that citizens have to engage with their community should perhaps be considered. Therefore, the different way of measuring civic engagement could have led to this difference in the results. These authors also highlight the importance of interpreting the results within the cultural context.

On the other hand, in relation to the Big Five traits, the association of civic engagement with openness to experience is the most prominent, being the trait with the highest association compared to agreeableness and conscientiousness (with no association with extraversion and neuroticism). In this case, H2 is also only partially accepted, as we expected to obtain significant associations with all five traits (positive for all except neuroticism), but civic engagement was only significantly (and positively) associated with three of the traits (i.e., agreeableness, conscientiousness, and openness to experience).

These results do not coincide with those obtained in previous literature, since no study has obtained the same associations with the same traits [6,11,12,13,14,15]. However, several studies have obtained positive associations with openness to experience, agreeableness, and conscientiousness (e.g., [11,15]). In the same way, there are also some studies that have not obtained associations with neuroticism [15], but all the articles already cited have obtained relationships with extraversion.

It is important to note that in the present study, the associations between civic engagement and the Big Five were also controlled for the association with the Dark Triad, which may have led to this discrepancy with the results of previous studies [7]. In addition, the correlations in this study did show significant relationships with the five traits, consistent with previous literature, e.g., [11]. Moreover, each study seems to look at civic engagement in less or more depth (asking more generally whether they are committed to the community or asking about more specific behaviors, such as voting, following the rules, participating in associations, etc.) and depending on this specificity level, different relationships have been obtained [6,11,12,13,14,15]. In our case, the questions were more general, which could cause participants to respond in more general terms, knowing that they do engage with their community by engaging in certain behaviors, but not others. Consequently, our results could be somewhat general.

Third, the regression model allowed us to test the last hypothesis of the paper (H3). In this case, H3 is accepted, since it was expected to obtain differences in the magnitudes of association with civic engagement when malevolent traits are associated alone and when they are associated with more general personality traits, and this is what was obtained. The contribution of all traits together (the Dark Triad together with the Big Five) being greater for civic engagement. Furthermore, when the Big Five are included in the model, the association with Machiavellianism and psychopathy is null and only narcissism (from the Dark Triad traits) is associated (and positively) with civic engagement. The association with the three dark traits is greater when they do so without the contribution of the Big Five.

These latter results cannot be compared with previous literature since this is the first study to analyze the association between civic engagement and personality in the same regression model, i.e., both the more general and the more malevolent traits. Perhaps, as Weinschenk [7] says, the association between some traits and others could explain the differences exposed in this work. However, it is important to consider that some authors consider the bias (in multivariate analyses) in the interpretations of the results problematic and some studies have concluded that the inclusion of the Dark Triad personality traits does not offer real predictive advantages over the HEXACO personality model, which contemplates the honesty–humility variable [37,38,39].

It is important to highlight the association obtained between civic engagement and narcissism (different from that obtained with the other two malevolent traits of the Dark Triad), as the results point to the fact that people with narcissistic traits do seem to engage with their community. Again, these results may be explained by the association between some traits and others, that is, by the relationship with general personality traits [7]. Moreover, the relationship between narcissism and the general traits has already been tested and in this study the same relationships have been found as in previous literature, i.e., positive relationships with extraversion, openness to experience, and conscientiousness, and negative ones with neuroticism and agreeableness [40,41,42,43,44,45,46,47].

These findings agree with the ones reported by Van Groningen et al. [22]. In their study, they concluded that, perhaps, general traits could be conferring narcissism the characteristic of being a protective factor with respect to the rest of the traits of the Dark Triad. However, it is important to mention the weak, albeit significant, correlation between narcissism and agreeableness, contrary to that found in previous literature (stronger correlation). This finding could be since the SD3 mainly measures the agentic components of narcissism, and not the antagonistic and vulnerable components, and it is likely that it is this agentic narcissism that correlates with civic engagement. Future research could explore this further [45,47,58].

These results highlight the need to study the subclinical narcissistic personality trait included in the Dark Triad. As with previous studies, this trait, despite being considered a malevolent personality trait, maintains a positive relationship with other variables considered socially desirable, such as emotional intelligence and well-being [21,22,23,24,25,26,27].

Another possible explanation for the relationship between civic engagement and narcissism is the one pointed out by Pruysers and colleagues [6]. They consider that people with narcissistic traits may seek praise and admiration from others and therefore perform good deeds (and thus perhaps also exhibit other desirable characteristics such as those mentioned above) [24,26,27].

Moreover, it is well known that people with narcissistic traits constantly need to boost their self-esteem and ego [18,59,60,61] and the positive relationship between having high collective self-esteem and participating in the community has also been seen [62]. Therefore, these people could perform these good acts, but without becoming excessively sympathetic and generous to others, which could explain the negative relationship obtained in this study and in previous literature (null in some studies) between narcissism and trait agreeableness [41,44,46].

### Limitations and Future Lines of Research

A possible limitation of this study is the difficulty in generalizing the results in terms of civic engagement. As has already been mentioned, depending on how this concept is treated (whether in a more general or more specific way), the results may vary and stronger or weaker relationships with other variables may be obtained. Therefore, as a future line of research, it might be interesting to compare these results using another questionnaire that measures civic engagement more specifically.

Another possible limitation of this study is that no internal consistency calculation is available for the instrument used to assess the Big Five personality traits, and the correlation between the two items of some of the factors in the present sample, especially of the agreeableness factor, is very low. Similarly, Cronbach’s alpha in the case of narcissism and psychopathy is not too high, which could indicate certain reliability problems.

A final limitation is, being a cross-sectional study, there is difficulty in generalizing the results and in establishing causality between variables. As a future line of research, we propose to analyze longitudinally the associations between civic engagement and general and malevolent personality traits.

## 5. Conclusions

This is the first study to jointly analyze (in the same regression model) the association of malevolent personality traits (Dark Triad) and the Big Five with civic engagement. It highlights, on the one hand, that the Dark Triad traits do associate with civic engagement, and, on the other hand, it highlights the specific contribution of narcissism and openness to experience (both positively associated). Following the indications of Van Groning [22] and Weinschenk [7], the positive relationship between narcissism and general personality traits could explain why people with narcissistic traits have a more favorable attitude towards civic behaviors, which points to a greater tendency towards good citizenship and greater civic engagement with their community.

However, it is important not to forget that these dark traits have been linked to a wide variety of violent behaviors (bullying, sextortion, intimate partner violence, and cyber violence or general delinquency, among others) [28,29,30,31,32,33], so perhaps, people with narcissistic traits perform civic behaviors in order to obtain their own benefits and reinforce their self-esteem [18,59,60,61]. In addition, it seems that people are more likely to participate in collective actions when these are related to their own interests [63].

This study provides further evidence of how the narcissistic personality trait differs from the other two malevolent traits that make up the Dark Triad. Given that these traits are also associated with maladaptive behaviors, as just discussed, knowing all the characteristics of these malevolent traits could facilitate the design of prevention programs aimed at reducing such maladaptive behaviors.

As future lines of research, we suggest the need to continue investigating the subclinical narcissistic trait, since it could be considered as a factor of self-protection against the other two traits of the Dark Triad (Machiavellianism and psychopathy). In addition, future research could further investigate the question of whether people with narcissistic traits are more likely to participate in collective actions because they seek their own benefits. In general, further analysis of all the relationships discussed in this study and establishing the causality of the different variables is encouraged.

## Figures and Tables

**Table 1:** Means and Standard Deviations for Civic Engagement, Dark Triad, and Big Five.

	Total (*N* = 1175)		
	Range of Scores	*M*	*SD*
Civic engagement	7–42	31.68	5.43
Big Five			
Extraversion	2–12	7.37	2.78
Agreeableness	2–12	7.96	2.06
Conscientiousness	2–12	7.54	2.10
Openness to experience	2–12	8.72	2.31
Neuroticism	2–12	7.07	2.57
Dark Triad			
Narcissism	0–36	15.11	5.28
Machiavellianism	0–36	16.77	6.60
Psychopathy	0–36	9.13	5.54

**Table 2:** Bivariate Correlations Among Study Variables.

	Civic Engagement	Extraversion	Agreeableness	Conscientiousness	Openness to Experience	Neuroticism
Civic engagement		0.22 *	0.14 *	0.23 *	0.28 *	−0.09 *
Narcissism	0.25 *	0.40 *	−0.09 *	0.14 *	0.15 *	−0.15 *
Machiavellianism	−0.11 *	−0.05	−0.29 *	−0.06	−0.05	−0.01
Psychopathy	−0.08	0.06	−0.22 *	−0.09 *	0.01	0.06

Note. * *p* < 0.0056 (Bonferroni fit).

**Table 3:** Associations between civic engagement, Dark Triad traits, and the Big Five personality traits.

		Block 1				Block 2				Block 3			
C V	P V	β	*t*	*r_x.y_*	*sr*²	β	*t*	*r_x.y_*	*sr*²	β	*t*	*r_x.y_*	*sr*²
C E	G	0.01	0.34	0.01	0.01%	0.02	0.80	0.02	0.05%	0.03	1.06	0.03	0.08%
	N					0.35	11.45 *	0.32	9.92%	0.23	7.05*	0.19	3.42%
	M					−0.16	−4.78 *	−0.13	1.74%	−0.09	−2.69	−0.07	0.49%
	P					−0.11	−3.21 *	−0.09	0.77%	−0.07	−2.14	−0.06	0.31%
	E									0.06	2.09	0.06	0.30%
	A									0.10	3.61 *	0.10	0.90%
	C									0.15	5.38 *	0.14	1.99%
	O									0.22	7.99 *	0.21	4.37%
	NE									−0.07	−2.42	−0.06	0.40%
	*R^2^*	0.01				0.11				0.19			
	*F*	0.12				36.83 *				32.48 *			

Note. CV = Criterion variable; PV = Predictor variable; CE = Civic engagement; G = Gender; N = Narcissism; M = Machiavellianism; P = Psychopathy; E = Extraversion; E = Agreeableness; C = Conscientiousness; O = Openness to experience; NE = Neuroticism; * *p* < 0.0056 (Bonferroni fit).

## Data Availability

The data presented in this study are openly available in the OSF repository at:10.17605/OSF.IO/TWUFH, https://osf.io/twufh/?view_only=f1ee58b3d8e4495bacc81ee7d1c72024, accessed on 30 September 2021.
